# Medial Open Wedge High tibial Osteotomy (MOWHTO) does not relevantly alter patellar kinematics: a cadaveric study

**DOI:** 10.1007/s00402-020-03578-1

**Published:** 2020-08-20

**Authors:** Felix Greimel, Guenther Maderbacher, Clemens Baier, Bernd Krieg, Florian Zeman, Joachim Grifka, Armin Keshmiri

**Affiliations:** 1 Department of Orthopedics, University Medical Center Regensburg, Asklepios Klinikum Bad Abbach, Kaiser-Karl-V.-Allee 3, 93077 Bad Abbach, Germany; 2 Center for Clinical Studies, University Medical Center of Regensburg, Franz-Josef-Strauss-Allee 11, 93053 Regensburg, Germany; 3 Orthopaedic Center in Helios, Helene-Weber-Allee 19, 80637 München, Germany

**Keywords:** Patellar kinematics, High tibial osteotomy, HTO, Patellar tracking, Computer navigation

## Abstract

**Purpose:**

The purpose of this study was to quantify the influence of medial open wedge high tibial osteotomy on patellar kinematics using optical computer navigation, as anterior knee pain infrequently occurs postoperatively and the reason is still being unknown.

**Methods:**

Ten medial open wedge high tibial osteotomies at supratuberosity level in 5 full body specimens were performed. The effect of the surgical procedure on patellar kinematics, measured at 5 and 10 degrees of leg alignment correction angle, was analyzed and compared to native patellar kinematics during passive motion—regarding patella shift, tilt, epicondylar distance and rotation. Linear mixed models were used for statistical analysis, a two‐sided *p* value of ≤ 0.05 was considered statistically significant.

**Results:**

Tilt behavior, medial shift and epicondylar distance did not show a significant difference regarding natural patellar kinematics at both osteotomy levels. Both osteotomy correction angles showed a significant less external rotation of the patella (*p* < 0.001, respectively) compared to natural kinematics.

**Conclusions:**

Except less external rotation of the patella, medial open wedge high tibial osteotomy does not seem to relevantly alter patellar alignment during passive motion. Future clinical studies have to prove the effect of MOWHTO on patellar kinematics measured in this experimental setup, especially regarding its influence on anterior knee pain.

## Introduction

High tibial osteotomy (HTO) is a widespread used, successful and continuously improving procedure in joint preserving orthopedic knee surgery [1]. Open and closed wedge osteotomies as well as valgisation and varisation techniques have been described. It is recognized to be a successful joint preserving procedure for unilateral knee osteoarthritis [1, 2]. Postoperative anterior knee pain is being reported to be one possible complication in the postoperative course [3]. It is described to be caused by patella height alterations often, e.g. patella infera [4–7]. HTO with a descending cut at the tibial tuberosity has been recently developed, as this procedure does not alter patellar height [8]. If and how classical open wedge HTO affects patellar kinematics remains unclear so far [6]. Furthermore, it is discussed if possible alterations might lead to a premature occurrence of patellofemoral osteoarthritis, the topic is, therefore, of great importance regarding the long-term clinical outcome [9, 10].

The purpose of the present experimental investigation was to assess the effect of medial high tibial open wedge osteotomy on patellar kinematics using an optical computer navigation system, as the effect of HTO on patellar kinematics remain unclear. The hypothesis was, that open-wedge HTO predisposes for a lateral patella shift and tilt, an increase of epicondylar distance and an outer rotation of the patella. To the authors' knowledge, this is the first survey quantifying and analyzing the influence of medial open wedge HTO on patellar kinematics using an optical computer navigation device.

## Material and methods

Ten Thiel-embalmed lower extremities, attached to the torso of five full cadaveric bodies, were used for this experimental investigation. All knees were free of osteoarthritic degeneration, dysplasia or altered lower limb alignment—based on the information of an x-ray and a CT scan in advance of the experiment—and had no prior surgical intervention or history of injury. Patellar kinematic (mediolateral shift, medial: + / lateral: − ; tilt, medial: − / lateral: + ; rotation, medial: + / /lateral: − ; epicondylar distance: distance between patella and anatomical transepicondylar axis) were analyzed using an optical computer navigation system (Knee Patella Tracking Software, BrainLAB; Feldkirchen, Germany) [11–13] before and after HTO with a leg alignment correction of 5° and 10°, respectively [14].

**Fig. 1:**
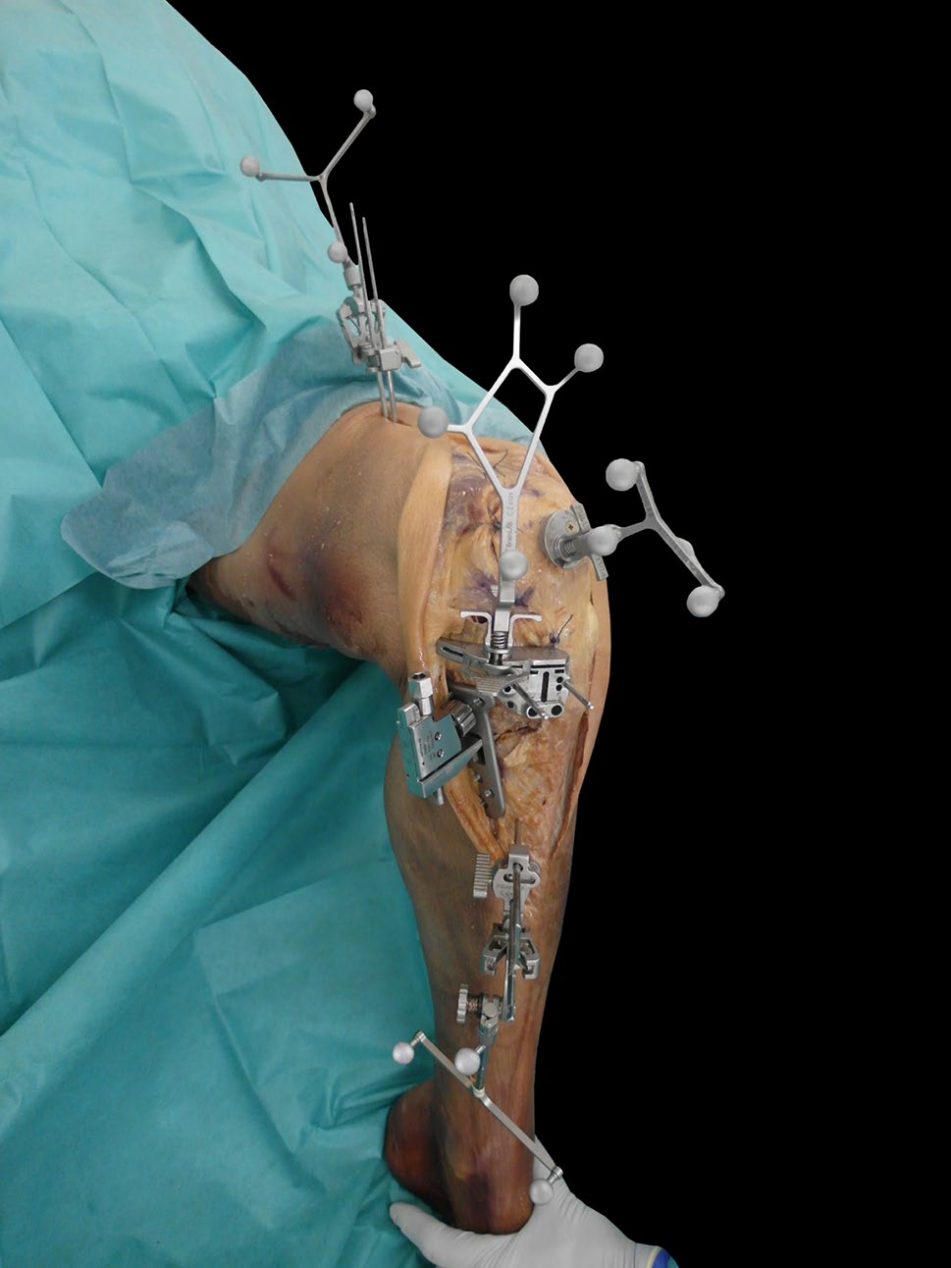
Experimental setup on full-body cadaver after preparation, referencing the optical navigation system and performing the osteotomy at 5 degrees of leg alignment correction, adjusted by the computer navigation

**Fig. 2:**
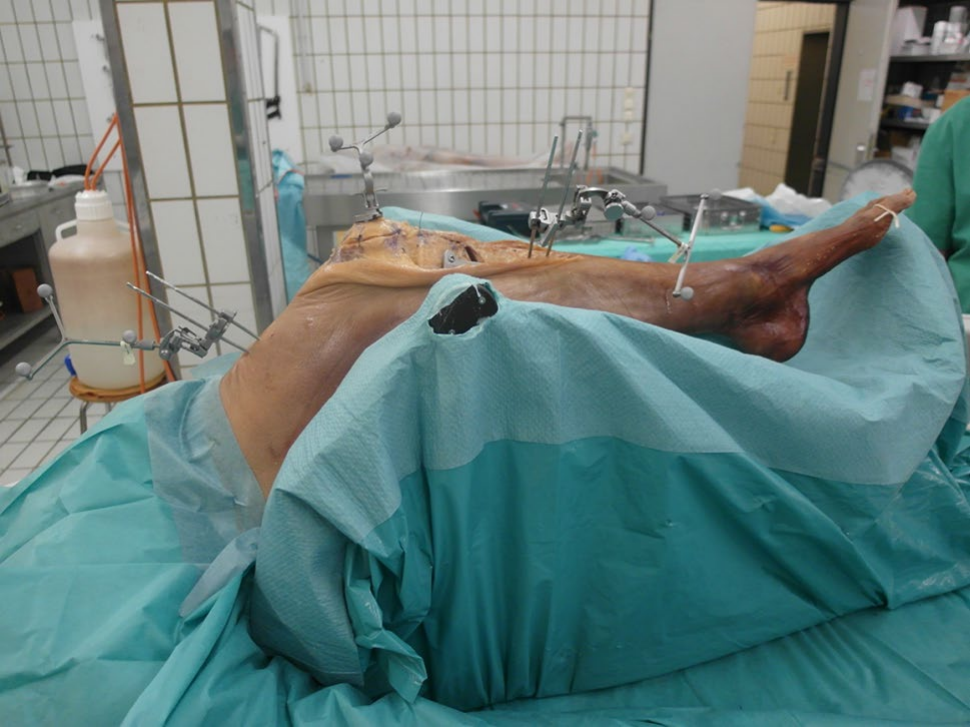
Experimental setup to assess patellar kinematics at different flexion angles on a continuous passive motion machine

After a standard median skin incision, a medial parapatellar approach was conducted. The knee joint capsule of each knee was marked at five standardized locations for later anatomic capsule closure. The reference arrays of the optical navigation system were attached to the reverse side of the patella (anterior), to the distal femur and to the proximal tibia with 2.5 mm bicortical bone pins, accordingly to the manufacturer’s recommendations. To avoid unnecessary parapatellar soft tissue tension while flexion, the femoral reference array was placed through an additional skin incision proximally to the knee approach. Landmarks for tibiofemoral and patellofemoral kinematics assumed by the navigation software were recorded. After the registration process, the knee capsule was closed carefully at the above mentioned standardized locations to attain anatomical aligned capsule closure. No gap occurred after closure of the capsule. Three motion cycles from 30 to 90 degrees of flexion were performed while placing the lower limbs on a continuous passive motion machine. Patellofemoral kinematics were recorded by the navigation system, a mean of the three motion cycles was calculated at every 10 degrees of flexion. Values from 10 to 30 degrees of flexion were irregular due to missing capsule tension and were excluded from the study protocol. The procedure was repeated after performing a classical medial open wedge high tibial and lateral inclined osteotomy at supratuberosity level with 5 and 10 degrees of leg alignment correction, respectively. The leg alignment correction was adjusted precisely by using the optical navigation system (Fig. 1). A conventional osteosynthesis plate was used to fixate the osteotomy during the experimental passive motion process (Fig. 2). All surgical procedures were performed by one of the authors to ensure comparable results.

According to our local ethical committee, IRB approval is not necessary.

### Statistical analysis

Mean and standard deviation of patellar kinematic parameters (shift, tilt, epicondylar distance, rotation) were calculated at 7 positions between 30 and 90 degrees of flexion in 10° steps. Data were normally distributed. Linear mixed models with cadaver as random effect and flexion of the knee, leg alignment and body side as fixed effects were used to assess the influence of leg alignment on patellar kinematic parameters. A two‐sided *p* value of ≤ 0.05 was considered statistically significant. Due to the exploratory nature of the study, no adjustments for multiple comparisons were made. All significant results have to be validated in further studies. Statistical analyses were performed with SAS 9.4 (SAS Institute, Cary NC).

## Results

Mean values and standard deviation of the parameters for the three test intervals native patella kinematics, patella kinematics at 5° and 10° leg alignment valgisation are presented in Table 1. Mean differences (estimated marginal means), 95%-Confidence Intervals and *p*-values are shown in Table 2.

**Table 1:** Mean values and standard deviation of patella kinematics at a range of 30 and 90 degrees of flexion

Flexion	30°	40°	50°	60°	70°	80°	90°
Epicond. distance (mm)							
Native	23.6 (5.4)	21.4 (5.6)	19.8 (5.5)	18.4 (5.1)	17.4 (4.5)	16.7 (3.9)	16.4 (3.3)
Osteotomy 5°	22.9 (5.8)	21.1 (5.7)	19.7 (5.4)	18.4 (4.8)	17.5 (4.1)	16.9 (3.4)	16.6 (2.9)
Osteotomy 10°	22.3 (5.8)	20.4 (5.8)	19.1 (5.4)	18.0 (4.9)	17.3 (4.1)	16.8 (3.4)	16.6 (3.0)
Tilt (°)							
Native	22.0 (11.3)	22.1 (11.1)	22.0 (10.8)	21.7 (10.3)	21.1 (10.0)	20.5 (9.7)	19.7 (9.5)
Osteotomy 5°	23.0 (10.4)	23.0 (10.1)	22.8 (9.8)	22.2 (9.6)	21.5 (9.3)	20.7 (9.2)	20.0 (9.0)
Osteotomy 10°	22.8 (10.7)	22.8 (10.4)	22.5 (10.1)	21.9 (10.0)	21.2 (9.8)	20.4 (9.5)	19.7 (9.5)
Rotation (°)							
Native	− 0.1 (14.8)	− 1.9 (14.8)	− 3.6 (14.8)	− 4.9 (14.9)	− 6.3 (15.1.)	− 7.2 (15.3)	− 7.7 (15.6)
Osteotomy 5°	1.4 (14.6)	− 0.2 (14.8)	− 1.7 (14.8)	− 3.0 (14.9)	− 4.3 (15.1)	− 5.2 (15.3)	− 5.6 (15.5)
Osteotomy 10°	2.3 (15.5)	0.6 (15.6)	− 0.8 (15.8)	− 2.2 (16.0)	− 3.2 (16.3)	− 3.8 (16.6)	− 4.0 (17.0)
ML-Shift (mm)							
Native	− 2.0 (6.5)	− 1.9 (6.4)	− 1.8 (6.1)	− 1.6 (6.0)	− 1.1 (5.6)	− 0.7 (5.6)	− 0.3 (5.6)
Osteotomy 5°	− 1.9 (5.7)	− 1.9 (5.9)	− 1.8 (5.6)	− 1.5 (5.4)	− 1.0 (5.4)	− 0.5 (5.4)	− 0.2 (5.5)
Osteotomy 10°	− 1.6 (5.6)	− 1.4 (5.3)	− 1.4 (5.3)	− 1.0 (5.0)	− 0.7 (5.1)	− 0.4 (5.4)	0.1 (5.4)

**Table 2:** Mean difference (estimated marginal means), *p*-values and 95%-confidence intervals (95%-CI) of patella kinematics at a range of 30 and 90 degrees of flexion

Flexion	Mean difference	*p*-values	95%-CI
Epicond. distance (mm)			
Native vs. osteotomy 5°	0.09	0.78 (ns)	− 0.54 to 0.73
Native vs. osteotomy 10°	0.44	0.17 (ns)	− 0.19 to 1.08
Osteotomy 5° vs. 10°	− 0.35	0.27 (ns)	− 0.99 to 0.28
Tilt (degrees)			
Native vs. Osteotomy 5°	− 0.58	0.14 (ns)	− 1.34 to 0.18
Native vs. Osteotomy 10°	− 0.31	0.43 (ns)	− 1.07 to 0.45
Osteotomy 5° vs. 10°	− 0.27	0.48 (ns)	− 1.03 to 0.49
Rotation (°)			
Native vs. osteotomy 5°	− 1.88	< 0.001	− 2.98 to − 0.79
Native vs. osteotomy 10°	− 2.96	< 0.001	− 4.05 to − 1.86
Osteotomy 5° vs. 10°	1.07	0.05 (ns)	− 0.02 to 2.17
ML-Shift (mm)			
Native vs. osteotomy 5°	− 0.09	0.782 (ns)	− 0.73 to 0.55
Native vs. osteotomy 10°	− 0.4429	0.173 (ns)	− 1.08 to 0.20
Osteotomy 5° vs. 10°	0.3529	0.277 (ns)	− 0.29 to 0.99

*ns*  not significant

### Epicondylar distance

**Fig. 3:**
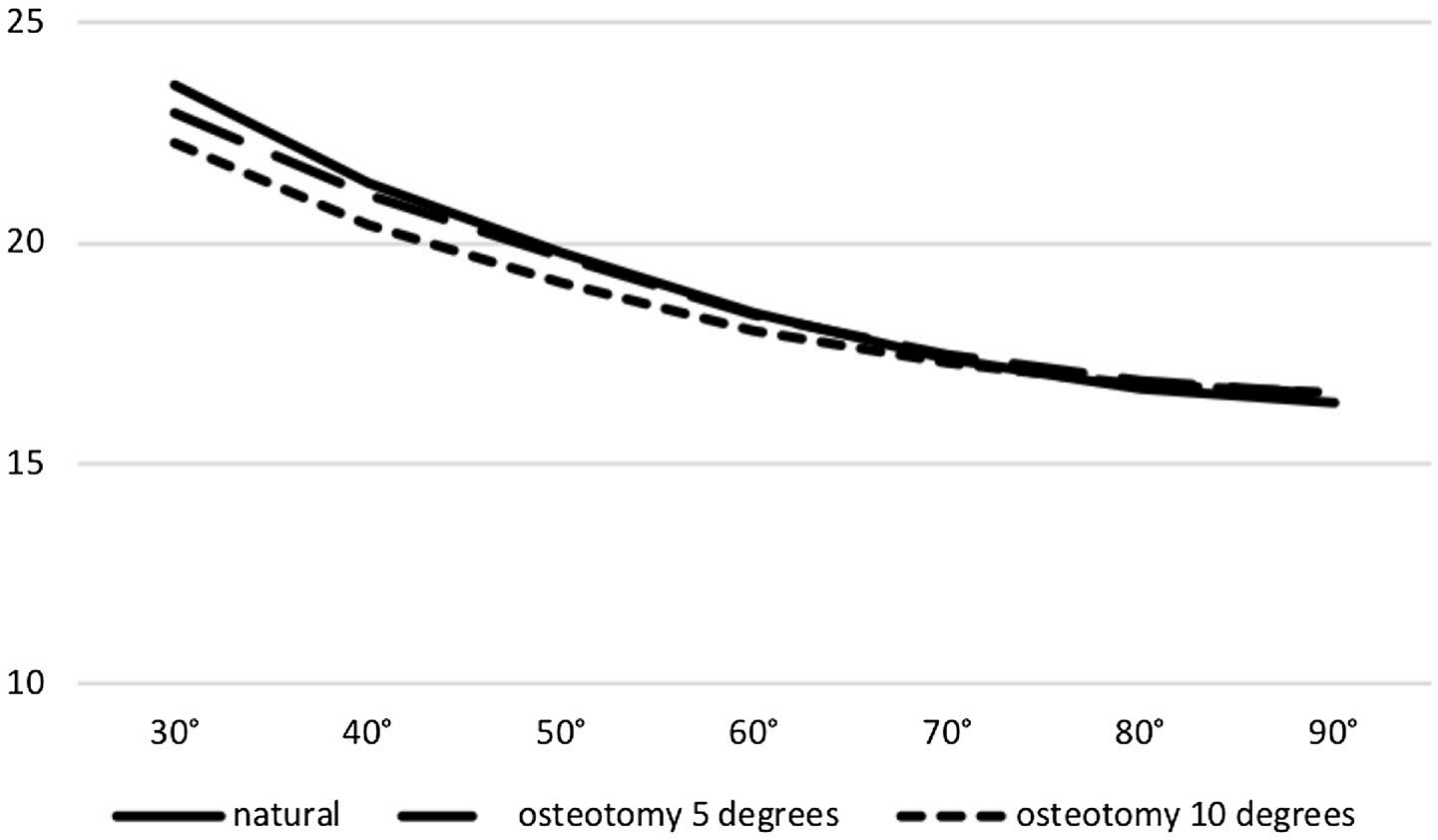
No significant difference between natural kinematics and osteotomy kinematics at 5 and 10 degrees of valgisation on patella epicondylar distance between 30 and 90 degrees of flexion (ns, respectively; *x*-axis: degrees of flexion; *y*-axis: distance in mm)

Epicondylar distance decreased during flexion in all groups. No statistically significant change for epicondylar distance between the native knee and both 5° and 10° valgisation could be stated (ns, respectively) for the overall range of motion (Fig. 3).

### Tilt

**Fig. 4:**
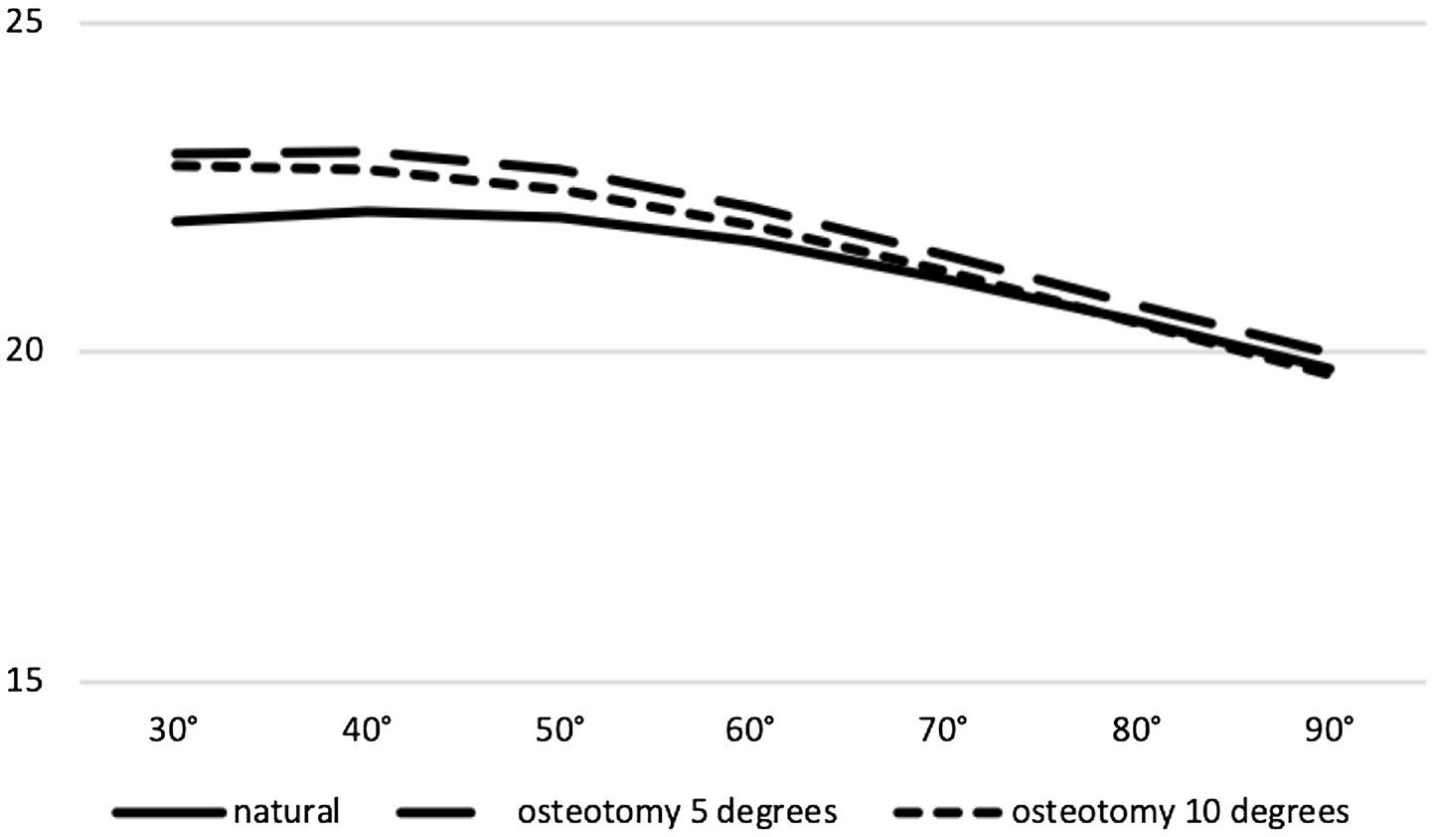
No significant difference between natural kinematics and osteotomy kinematics at 5 and 10 degrees of valgisation on patella tilt between 30 and 90 degrees of flexion (ns, respectively; lateral tilt in degrees: + ; *x*-axis: degrees of flexion; *y*-axis: tilt in degrees)

Lateral patellar tilt showed a continuous decrease from 30 to 90 degrees of flexion in all groups. No significant difference between the native patellar tilt and the intervention groups could be demonstrated at 5 and 10 degrees of correction (ns, respectively) (Fig. 4).

### Shift

**Fig. 5:**
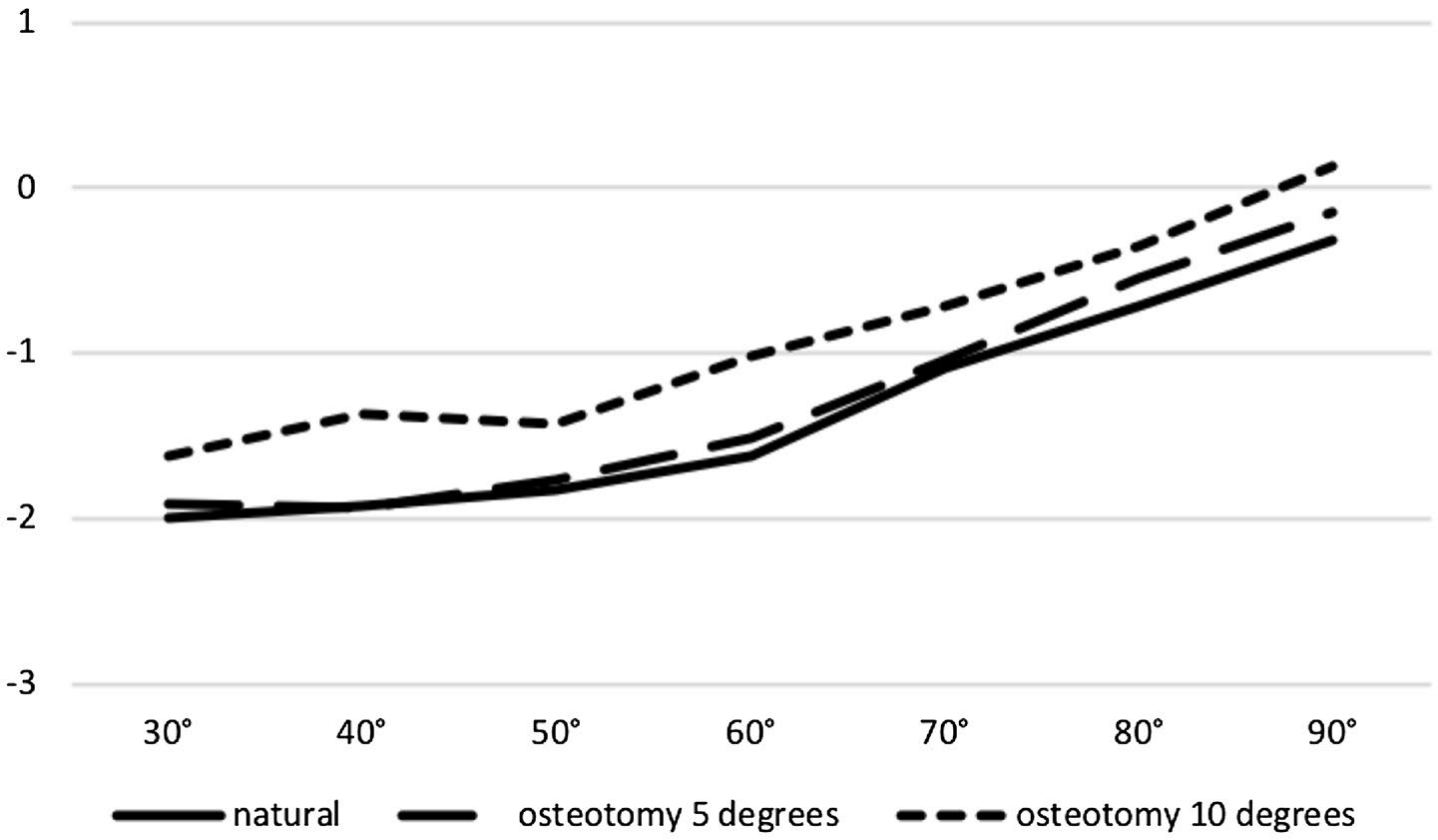
No significant difference between natural kinematics and osteotomy kinematics at 5 and 10 degrees of valgisation on patella shift between 30 and 90 degrees of flexion (ns, respectively; medial shift in mm: + ; *x*-axis: degrees of flexion; *y*-axis: shift in mm)

A progressive medial shift of the patella up to 90 degrees of flexion was observed in the native knee and at both osteotomy levels. Both 5° and 10° osteotomy groups could restore the mediolateral shift of the intact knee (ns, respectively) (Fig. 5).

### Rotation

**Fig. 6:**
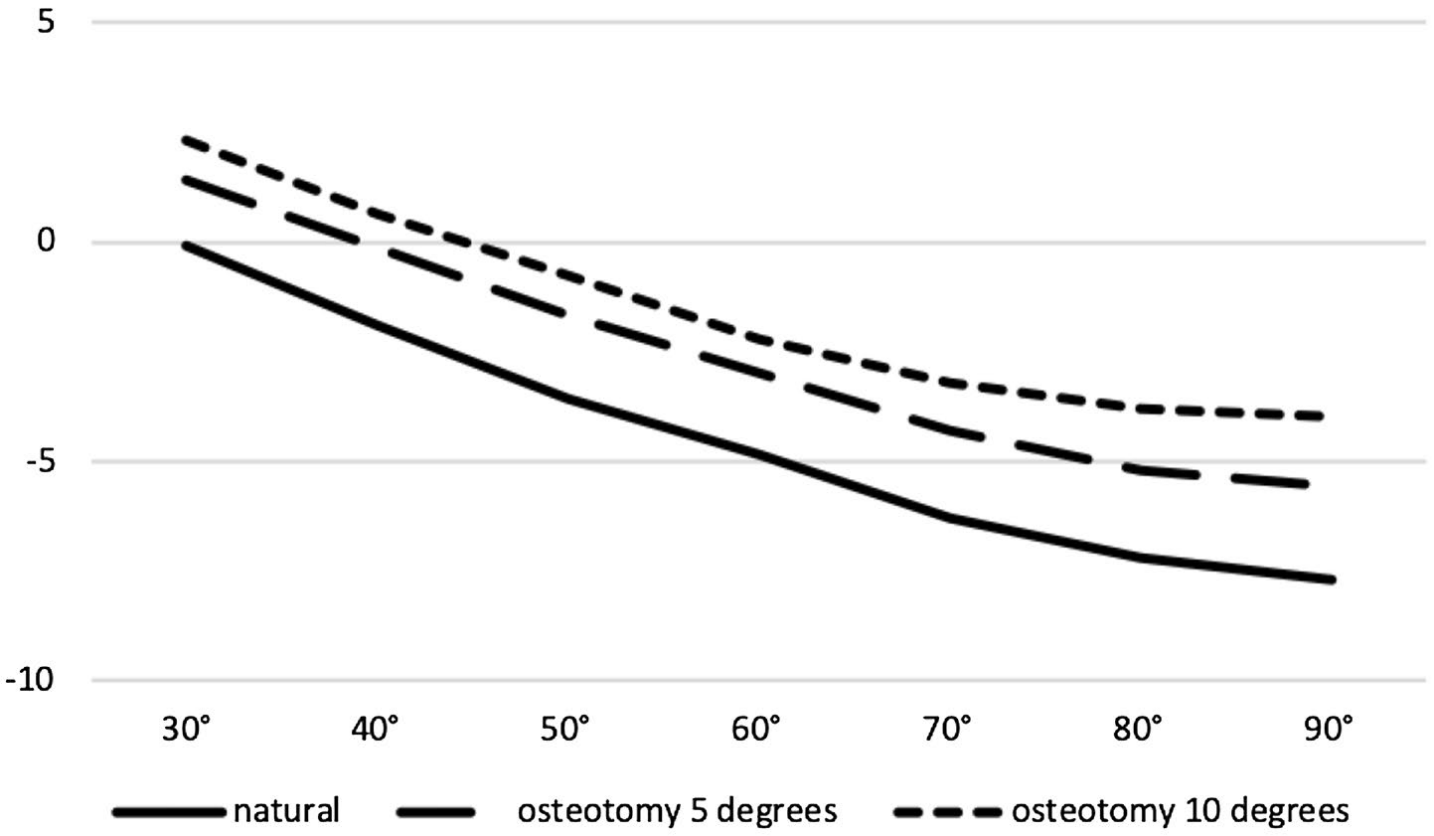
Significant difference between natural kinematics and kinematics at both osteotomy levels (5 and 10 degrees of valgisation) on patella rotation at all 10° steps between 30 and 90 degrees of flexion (*p* < 0.001, respectively; outer rotation in degrees: –; *x*-axis: degrees of flexion; *y*-axis: degrees of rotation)

The patella showed a continuous external rotation with increased flexion in all groups. Both osteotomy corrections levels led to a significantly less external rotation of the patella during flexion cycle (*p* < 0.001, respectively) (Fig. 6).

## Discussion

This investigation was performed to assess patellar tracking alterations after medial open wedge high tibial osteotomy in an experimental setup. The most important finding of this study is that patellar kinematics do not seem to be relevantly altered by medial open wedge high tibial osteotomy regarding medial shift, lateral patellar tilt and epicondylar distance in a cadaveric setup. Intraoperative patellar kinematics after HTO seem to be secondary for the surgeon at first sight. Anterior knee pain is being reported to be as high as 11.4% [4–7, 10] and intraoperative patellar maltracking might be a possible reason for its occurrence in the postoperative course. Furthermore, the success of the surgical procedure at the medium and long term and the possibility of development of patellofemoral osteoarthritis might be affected by patellar kinematics, therefore, the question in dispute is of potentially great importance [9, 10, 15].

We are able to demonstrate that natural patellar kinematics can be preserved by medial open wedge HTO within the evaluated range of motion, except stating less external rotation of the patella in this experimental setup.

The reason for less external rotation of the patella during flexion remains unclear. One possible explanation of the authors is that after performing the open wedge in a valgisation direction, the lateral patellar retinaculum will possibly relieve tension, and, therefore, the patella could experience the observed internal rotation movement compared to natural patella kinematics. If this observation of slightly less outer rotation at all flexion degrees is clinically relevant remains ambiguous, since no evaluation on this topic is available in the literature to the authors knowledge. Changes in moment arm due to rotation alteration might play a role and has to be assessed in the future. Still, all other, to our opinion more important, parameters epicondylar distance, tilt and medio-lateral shift did not show a statically significant difference at all times and, therefore, punctuates the small influence of HTO on patellar kinematics in this experimental survey.

Parts of the findings of the present investigation confirm the results of Lee et al. [16], who stated that patellar tilt and shift were not altered by medial open wedge HTO in 46 knees, although the results were not measured intraoperatively, but postoperatively at a mean of 44 months on X-rays. Epicondylar distance and patellar rotation could not be measured.

Similar results were reported by Yang et al. [17], where patellar shift and tilt did not differ significantly in 61 knees compared to natural patellar kinematics, measured on X-rays postoperatively. Again, epicondylar distance and patellar rotation were not measured.

Javidan et al. [18] reported a significant increase of patellofemoral peak pressure at various degrees of flexion on nine human cadaveric knees after medial wedge HTO of 10 mm in comparison with the native knee, which can possibly cause anterior knee pain or the occurrence of patellofemoral osteoarthritis in the postoperative course. We are not able to find changes in epicondylar distance, shift and, tilt, and therefore contact stress is unlikely to be altered. Therefore, the findings of Javidan et al. cannot be supported by the present investigation. However, explicit contact forces in the patellofemoral joint could not be measured with the technique of optical navigation.

Bito et al. [19] described affection of patellar tilt and patellar height after performing medial open wedge HTO in 49 cases, while the patellar shift was not altered—again measured on X-rays postoperatively. They summarized that medial open wedge HTO negatively affects the congruency of the patellofemoral joint. These findings, except patellar height, which was not evaluated in the present study, are therefore in contrast to the findings of the present investigation. The topic of patellofemoral contact stress is, furthermore, contrarily discussed in the literature. Stoffel et al. [20] reported no alteration of patellofemoral contact stress in 2 medial open wedge HTOs performed on human cadaveric specimens.

Amis et al. described in a review of the literature on the topic, that there is little evidence regarding many of the accepted surgical principles when performing HTO and its effect on knee kinematics and secondary effects such as alteration of collateral ligament tension or of the height of the patella [21]. Ishimatsu et al. compared a hybrid closed wedge HTO and medial open wedge HTO technique on 52 knees and found that hybrid closed wedge HTO led to improved patellofemoral joint congruency, while mid-term clinical outcomes were the same. Kloos et al. showed that HTO with a proximal biplanar osteotomy of the tuberositas tibia significantly increases patellofemoral pressure conditions depending on the correction angle in a cadaveric experiment [22]. In contrast, they described a distally directed biplanar osteotomy to diminish these effects while implantation of an extracapsular, extra-articular absorber showed no influence on the patellofemoral compartment at all. They proposed that patients with varus alignment with additional retropatellar chondropathia should be treated with a distally adverted osteotomy to avoid further undesirable pressure elevation in the patellofemoral joint. Tanaka et al. performed a clinical level IV survey after classical medial open wedge HTO within 52 knees and summarized, that Cartilage injuries in patellofemoral joints tended to progress after open wedge HTO in patients with an increase of the medial proximal tibial angle of more than 9° [23]. They proposed that other type of surgery may need to be considered to avoid the early progression of patellofemoral cartilage injuries. Otsuki et al. performed a clinical level III study after hybrid HTO or open wedge HTO in 48 cases and summarized that hybrid HTO provides a better post-operative patellofemoral joint than open wedge HTO with regards to patellar position and reduction of the TT–TG distance, as well as improved clinical outcomes [3]. Another level IV study of Krause et al. described that a descending HTO technique did not influence patella height or increase the posterior tibial slope compared to a biplanar ascending medial open-wedge HTO [24]. Otsuki et al. performed another level III study to analyze the correlation between varus knee alignment and patellofemoral osteoarthritis. They concluded that patellar tilt and the TT–TG distance are considered to be critical factors for the severity of patellofemoral osteoarthritis [25]. Understanding the critical factors for patellofemoral osteoarthritis in varus knees such as the TT–TG distance and patellar tilt might facilitate the prevention of patellofemoral osteoarthritis using procedures such as high tibial osteotomy. In the end, we still do not know the HTO technique of superiority and their effects on patellofemoral kinematics and progress of patellofemoral osteoarthritis, regarding the present literature. In the present survey, only supratuberosity level medial open wedge HTO were performed, to receive comparable and statistical valuable results in this cadaveric investigation.

This study has several limitations. First, this study is of experimental nature conducted on Thiel-embalmed cadavers. The cadaveric specimens might not represent the natural kinematics of a knee from a living human performing a flexion cycle actively with all its muscle activity. However, this investigation was performed to simulate intraoperative surgical conditions using passive knee motion, therefore, the lower limbs were still attached to the torso of the cadaver. Relative changes were measured at three dedicated times intraoperatively (natural patellar kinematics, patellar kinematics at 5 and 10 degrees of valgisation), so that absolute differences were not relevant. To avoid bias in the motion cycles, a continuous passive motion machine was used. Furthermore, the investigated cadaveric knees were free from severe osteoarthritis or contractions. Furthermore, the specimen did not show isolated unicompartmental medial osteoarthritis, the classical indication for the investigated procedure.

In addition, an optical computer navigation device with an additional patella reference array was used. Optical computer navigation has been verified to be a reliable measurement tool to evaluate three-dimensional knee kinematics [26, 27] and relative values and kinematic changes were measured. However, the patella reference array theoretically might have affected patellar kinematics caused by its own weight, dynamics and center of gravity.

Furthermore, and although closure and reopening of the knee joint capsule were performed with great care and the fact that standardized locations for suturing the capsule were used, deviations in kinematic measured in comparison to intact knees might have occurred. As no capsule deterioration during flexion cycle was observed, this possible bias is unlikely to have affected our experimental results.

In addition, patellar kinematics were measured without muscle force and through passive range of motion on a passive motion machine, reflecting intraoperative conditions. However, data was collected using cadaveric knees still attached to the torso. Moreover, in a radiological investigation, Masri and McCormack reported, that quadriceps contraction does not alter the congruence angles obtained in 30° and 45° axial views [28]. Additionally, Grassi et al. showed, that there were no differences in knee kinematics between active and passive flexion–extension movement cycles regarding intra-operative kinematic analysis [29]. Therefore, our results should reflect on comparable native patellar tracking in this experimental setup. On the other hand, Ahmed et al. described that patellar tracking pattern—patellar shift, tilt and rotation—is controlled by passive restraint provided by the topographic interaction of the patellofemoral contacting surfaces while flexion of the knee between 30° to 100° in a biomechanical analysis [30]. Patellar medial–lateral translation was found to be controlled dominantly by the trochlear topography, while retropatellar topography also had a significant role in the control of the other two displacements. Heegaard et al. examined native patellar kinematics using a three-dimensional computer model based on a finite element method with regard to the joint's kinematics, associated tendinous and ligamentous forces, articular contact pressures occurring in the joint during its motion [31]. They described a wide range of variations of the contact pressure acting on the patella during knee flexion, differing from other surveys on patellar kinematics not considering soft tissue forces. Still, it was a theoretical model with all its in vitro limitations. Thus, our survey might have underestimated the effect of patellar tracking changes after HTO due to its limitation on 30° to 90° of knee flexion.

In the present investigation, cadaveric knees were used for the first time to simulate intraoperative conditions using an imageless navigation system in healthy knees to evaluate the effect of HTO on patellar kinematics.

This data can be used to perform clinical studies using navigation systems while performing HTO.

Clinical relevance can be drawn from the fact that neither the 5 mm nor the 10 mm medial open wedge HTO alters patellar kinematics relevantly in this experimental survey. The appearance of anterior knee pain after performing an open wedge HTO is unlikely to be caused by altered patellar kinematics, but possibly by patella height alterations, which is being supported in the current literature. This has to be investigated in detail in further clinical studies.

## Conclusions

The findings observed in this experimental setup show that natural patellar kinematics can be preserved by medial open wedge HTO interventions, except stating less external rotation of the patella, between 30° and 90° of flexion, in a passive motion cadaveric survey.

Anterior knee pain after HTO procedures, possibly influenced by patellar kinematic alterations, is unlikely to be explained by patella kinematic changes, but has to be investigated in future clinical studies.
