# Correction to: Medial Open Wedge High tibial Osteotomy (MOWHTO) does not relevantly alter patellar kinematics: a cadaveric study

**DOI:** 10.1007/s00402-021-04217-z

**Published:** 2021-10-16

**Authors:** Felix Greimel, Guenther Maderbacher, Clemens Baier, Bernd Krieg, Florian Zeman, Joachim Grifka, Armin Keshmiri

**Affiliations:** 1 Department of Orthopedics, University Medical Center Regensburg, Asklepios Klinikum Bad Abbach, Kaiser-Karl-V.-Allee 3, 93077 Bad Abbach, Germany; 2 Center for Clinical Studies, University Medical Center of Regensburg, Franz-Josef-Strauss-Allee 11, 93053 Regensburg, Germany; 3 Orthopaedic Center in Helios, Helene-Weber-Allee 19, 80637 München, Germany

## Correction to: Archives of Orthopaedic and Trauma Surgery 10.1007/s00402-020-03578-1

The article Medial Open Wedge High tibial Osteotomy (MOWHTO) does not relevantly alter patellar kinematics: a cadaveric study, written by Felix Greimel, Guenther Maderbacher, Clemens Baier, Bernd Krieg, Florian Zeman, Joachim Grifka and Armin Keshmiri, was originally published electronically on the publisher’s internet portal on 20 August 2020 without open access. With the author(s)’ decision to opt for Open Choice the copyright of the article changed on 30 September 2021 to © The Author(s) 2021 and this article is licensed under a Creative Commons Attribution 4.0 International License, which permits use, sharing, adaptation, distribution and reproduction.

in any medium or format, as long as you give appropriate credit to the original author(s) and the source, provide a link to the Creative Commons licence, and indicate if changes were made. The images or other third party material in this article are included in the article’s Creative Commons.

licence, unless indicated otherwise in a credit line to the material. If material is not included in the article’s Creative Commons licence and your intended use is not permitted by statutory regulation or exceeds the permitted use, you will need to obtain permission directly from the copyright holder.

To view a copy of this licence, visit http://creativecommons.org/licenses/by/4.0/.

Open Access funding enabled and organized by Projekt DEAL.

The original article has been corrected.

